# Comparison of epidemiologic factors and eye manifestations of twin children with controls

**DOI:** 10.1186/s12886-023-02983-5

**Published:** 2023-06-01

**Authors:** Zhale Rajavi, Hamideh Sabbaghi, Reza Hasani, Narges Behradfar, Saeid Abdi, Bahareh Kheiri, Azadeh Haseli-Mofrad

**Affiliations:** 1 Negah Aref Ophthalmic Research Center, Shahid Beheshti University of Medical Sciences, Tehran, Iran; 2 Department of Ophthalmology, School of Medicine, Shahid Beheshti University of Medical Sciences, Tehran, Iran; 3 Ophthalmic Epidemiology Research Center, Research Institute for Ophthalmology and Vision Science, Shahid Beheshti University of Medical Sciences, 23 Paidar Fard, Bostan 9, Pasdaran Ave, Tehran, 16666 Iran; 4 Department of Optometry, School of Rehabilitation, Shahid Beheshti University of Medical Sciences, Tehran, Iran; 5 School of Medicine, Shahid Beheshti University of Medical Sciences, Tehran, Iran; 6 Ophthalmic Research Center, Research Institute for Ophthalmology and Vision Science, Shahid Beheshti University of Medical Sciences, Tehran, Iran

**Keywords:** Epidemiologic factors, Eye manifestations, Twins

## Abstract

**Purpose:**

The present study was aimed to compare the epidemiological and ocular findings of twin children in comparison with non- twin age matched individuals as their control.

**Methods:**

In this cross sectional study, a total of 90 twins (180 cases) were compared with 182 non- twin matched children. All the study participants were examined by a comprehensive ophthalmic examination including measurement of the best corrected visual acuity (BCVA), cycloplegic refraction, ocular deviation, strabismus as well as the anterior and posterior ophthalmic examinations. Demographic information of children were collected by using an organized questionnaire. Monozygotic twins were considered if there were similarity of their phenotypic characteristics and gender, otherwise the twins were considered as dizygotic.

**Results:**

The mirror- image twins (MIT) was defined according to the laterality of symmetrical ocular characteristics of twins. In this study, the mean age of the study participants was 7.08±4.42 and 7.58±3.99 years in twins and non-twins groups, respectively (*P*=0.253). Among the twins, 27 (30%) were monozygotic. Refractive form of MIT was seen in 5 twins (2.8%). The spherical refractive error was more hyperopic in twins compared to non- twins (*P*=0.041). BCVA in the twin group (0.07±0.16LogMAR) was significantly worse than non-twins (0.03±0.08LogMAR, *P* < 0.001) and higher percentage of them were amblyopic (37.2% versus 10.4%, *P*=0.005). Twin and controls had strabismus in 17.2% and 1.6%, respectively (*P* < 0.001). Regarding the comparison between mono- and dizygotic twins, more significant percentage of monozygotic twins had amblyopia (*P*=0.004) and strabismus (*P*=0.047). Multivariate analysis showed significant correlation among low gestational age and female gender, low birth weight and seizure.

**Conclusion:**

Female sex, less gestational age, low birth weight, amblyopia and strabismus were significantly higher in twins. Therefore, it is important to check their refractive error, amblyopia and strabismus to prevent their further complications.

## Introduction

Genetic and environmental factors have substantial roles in child developing and manifestation of various ocular features such as refractive errors and ocular deviations as well as congenital anomalies particularly in monozygotic twins [1]. According to the epidemiologic studies, the incidence of identical twin is estimated to be about 2 neonates per 1000 births in general population and between 8 to 30% among twin population [1, 2]. The mirror- image twins (MIT) phenomenon can be identified in 25% of the identical twins [1, 3, 4]. Each identical twins develops from a fertilized ovum with the same genetic and sexual characteristics, therefore similar phenotypic features would be expected [5]. In MIT phenomenon, both twins have similar characteristics but asymmetric, for instance, high hyperopia in the right eye of one child, whereas it can be identified in the left eye of the other child of one twin [1, 6].

The twin registry in California reported the incidence of congenital anomalies in 38/1000 children among 20803 twin pairs (3.8%) and there were a significant association of spina bifida and ocular deviations with a history of parental smoking [7]. Additionally, higher association was reported in monozygotic twins compared with dizygotic ones regarding different ocular parameters including cup/disc ratio [8], pupil size variation after dilating eye drops [9], anterior chamber depth [7], intraocular pressure [10] and corneal thickness [11]. In the study by He et al. [8], it was found that the refractive errors were correlated to genetic findings and it was not directly related to the environmental factors. Whereas, Cadet et al. [12] conducted a study on esotropic monozygotic twins with different age of strabismus onset and concluded that the environmental factors can have influential role on the visual function of strabismic patients regardless of genetic variations. Therefore, it was found that the refractive status can be determinated based on the both genetic and environmental factors.

In this study, we aimed to compare the epidemiological and ocular findings of twin children with non- twin ones as their control and we also desired to compare the corresponding factors between the monozygotic and dizygotic twins who were referred to Imam Hossein, Torfeh and Negah Eye centers.

## Methods

### Study method

In this cross sectional study, a total of 180 twins were compared with 182 non- twin children. Controls were matched with cases according to their age.

### Ethical considerations

An informed consent letter was obtained from all adult participants or their parents or legal guardian(s) if they were less than 18 years old after complete explanation of the study purpose. This study was approved by the Ethics Committee of Ophthalmic Research Center affiliated to SBMU using the approval number of IR.SBMU.ORC.REC.1398.026.

### Inclusion & exclusion criteria

All twin children were sequentially included and then age matched controls were participated. The in vitro fertilization and those with a history of retinopathy of prematurity were excluded from this study.

Monozygotic twins were considered if there were similarity of their phenotypic characteristics and sex [5]. Furthermore, the mirror- image twins (MIT) phenomenon was defined based on the laterality of symmetrical ocular characteristics of twins, for instance high hyperopia was identified in the right eye of one child, while it was detected in the left eye of the other child [1, 6].

### Examinations

Cycloplegic refraction was checked after 30-45 min following instillation of both tropicamide 1% and cyclopentolate 1% eye drops with 5 min apart. Best corrected visual acuity (BCVA) was measured using the linear Snellen E- chart at a distance of 6 m. Amblyopia was defined in cases with the monocular BCVA of 0.3 LogMAR or worse or it was considered if the difference of BCVA between the two eyes was at least two lines [13]. Ocular motility was tested in 9 cardinal visual gazes from + 4 to -4 as maximum overaction to maximum underaction, respectively. Ocular alignment was checked by either alternate prism cover test in cooperative or Krimsky method in non-cooperative children at both far (6 m) and near (33 cm) distances. All children who had a history of strabismus surgery or showed eye deviation were considered as strabismic cases. Examination of both anterior and posterior ocular segments were performed using slit lamp and indirect ophthalmoscope, respectively.

Additionally, an organized questionnaire was filled out for all participants to complete basic information including gestational age, birth weight (prematurity was considered if the birth weight of ≤ 1500gr and gestational age of ≤ 32 weeks) [14], systemic diseases, neurological disorders such as seizure as well as previous ocular surgery.

### Statistical analysis

To describe data, we used frequency (percent) and mean ± standard deviation. To evaluate the difference between two groups, we used Chi-Square and Fisher exact tests. Also, independent T- test was applied for comparison of the visual acuity outcomes between the two groups. Additionally, binary logistic analysis was applied to evaluate the effect of variables on gestational age. A P-value less than 0.05 was considered as statistically significant. All statistical analyses were performed using SPSS software (IBM Corp. Released 2016. IBM SPSS Statistics for Windows, Version 25.0. Armonk, NY: IBM Corp.).

## Results

In this cross sectional study, the ocular characteristics of a total of 90 twins (180 cases) were compared with 182 non- twin children. The mean age of the study subjects was 7.08 ± 4.42 and 7.58 ± 3.99 in the twins and non- twins groups, respectively (*P* = 0.253). Among the twins, 63 (70%) were dizygotic and 27 (30%) were monozygotic.

Demographic characteristics of subjects in the both monozygotic and dizygotic twins as well as non- twins are summarized in Table 1. As shown, incomplete gestational age, low birth weight, neonatal jaundice, history of being in incubator, female sex as well as history of strabismus surgery had higher rate in the twins group. Regarding the comparison between mono- and dizygotic twins, greater percentage of positive family history of twins was observable among dizygotic twins (*P* < 0.001).

**Table 1:** Demographic characteristics of the study subjects

Parameters	Levels	Groups					*P*-value
		Twins (*n* = 180)				Non- twins (*n* = 182)	
		Monozygotic (*n* = 54)	Dizygotic (*n* = 126)	*P*-value	Total (180)		
Sex (%)	Female	36 (66.7%)	72 (57.1%)	0.232**	108 (60.0%)	84 (46.2%)	0.01**
	Male	18 (33.3%)	54 (42.9%)		72 (40.0%)	98 (53.8%)	
Age	mean ± SD	7.44 ± 3.71	6.94 ± 4.65	0.478***	7.08 ± 4.42	7.58 ± 3.99	0.253***
	median (Range)	7 (1 to 14)	7 (1 to 20)		7 (1 to 20)	7 (1 to 16)	
Incomplete Gestational age		17 (31.5%)	39 (30.9%)	0.825**	56 (31.1%)	14 (7.7%)	< 0.001**
Low Birth weight (< 1500gr)		9 (16.7%)	13 (10.3%)	0.233**	22 (12.2%)	5 (2.7%)	0.001**
H/O Neonatal jaundice		25 (46.3%)	78 (61.9%)	0.052**	103 (57.2%)	63 (34.6%)	< 0.001**
H/O Seizure		2 (3.7%)	4 (3.2%)	0.864*	6 (3.3%)	4 (2.2%)	0.54*
H/O Incubator		31 (57.4%)	74 (58.7%)	0.777**	105 (58.3%)	7 (3.8%)	< 0.001**
Ocular anomaly	Ant. seg. anomaly	0 (0.0%)	2 (1.6%)	0.354*	2 (1.1%)	0 (0.0%)	0.62*
	Post. seg. anomaly	0 (0.0%)	0 (0.0%)		0 (0.0%)	1 (0.5%)	
H/O strabismus surgery		10 (18.5%)	16 (12.7%)	0.426**	26 (14.5%)	3 (1.6%)	< 0.001**
Twins in close family		19 (35.2%)	88 (69.8%)	< 0.001*	107 (59.4%)	-	-
Neurological delay		2 (3.7%)	4 (3.2%)	0.864*	6 (3.3%)	4 (2.2%)	0.536*
Physical delay		4 (7.4%)	3 (2.4%)	0.113**	7 (3.9%)	4 (2.2%)	0.38**

*MZ* monozygotic, *DZ* dizygotic, *M* male, *F* female, *Ant* anterior, *Post* posterior, *seg* segment, *wks* weeks, *gr* gram, *H/O* history of, *UA* unavailable, *n* number, *P* probability

^*^Based on Fisher exact test

^**^Based on Chi-square test

^***^ Based on T-test

Visual and ocular findings of both groups are presented in Table 2. The mean value of hyperopia was higher in twin children compared to non- twin individuals (*P* = 0.041). Ansiometropia and astigmatism were similar in the both groups and refractive form of MIT phenomenon was seen in 5 twins (2.8%). Best corrected visual acuity (BCVA) in the twins group was significantly less than non- twins (*P* = 0.001) and higher percent of them were amblyopic (*P* = 0.005). Furthermore, higher percentage of strabismus was identified in the twins group (*P* < 0.001). No statistically significant difference was observed in other variables between these two studied groups. Additionally, the extraocular muscles function was normal in both groups. Furthermore, more significant percentage of monozygotic twins had amblyopia (57.4% vs. 28.6%; *P* = 0.004) and strabismus (26% vs. 13.5%; *P* = 0.047) compared to the dizygotic twins. No statistically significant difference was identified between mono- and dizygotic twins in terms of refractive errors, MIT, BCVA, amblyopia, IOOA, pattern and ocular anomalies.

**Table 2:** Visual and ocular findings of the study subjects

Parameters	Levels	Groups					*P*-value
		Twins (*n* = 180)				Non- twins (*n* = 182)	
		Monozygotic (*n* = 54)	Dizygotic (*n* = 126)	*P*-value	Total		
SE (D)	Mean ± SD	0.976 ± 1.91	0.789 ± 1.97	0.407¥	0.819 ± 1.963	0.49 ± 2.33	0.041¥
	Median (Range)	0.94 (-3.00 to 8.37)	1.00 (-9.50 to 7.62)		0.875 (-9.5 to 8.37)	0.75 (-12.0 to 8.0)	
Anisometropia (SE ≥ 1.50D)	Yes	3 (5.5%)	4 (3.2%)	0.904**	7 (3.9%)	13 (7.1%)	0.25**
High Anisometropia (SE ≥ 3.00D)	Yes	1 (1.8%)	2 (1.6%)	0.455*	3 (1.7%)	2 (1.1%)	0.67*
Astigmatism (%)	WTR	68 (62.9%)	160 (63.5%)	0.552*	228 (63.4%)	201 (55.2%)	0.25*
	ATR	4 (4.4%)	6 (2.4%)		10 (2.7%)	17 (4.7%)	
	Oblique	2 (1.7%)	2 (0.8%)		4 (1.1%)	4 (1.1%)	
	No Astigmatism	34 (31.0)	84 (33.3%)		118 (32.8%)	142 (39.0%)	
Mirror Image of refractive error (%)		3 (5.6%)	2 (1.6%)	0.32*	5 (2.8%)	-	-
BCVA (LogMAR)	Mean ± SD	0.07 ± 0.16	0.06 ± 0.14	0.557¥	0.07 ± 0.16	0.03 ± 0.08	< 0.001¥
	Median (Range)	0.0 (0.0 to 1.0)	0.0 (-0.12 to 1.0)		0 (-0.12 to 1)	0 (0 to 0.7)	
Amblyopia (%)	Yes	31 (57.4%)	36 (28.6%)	0.004**	67 (37.2%)	19 (10.4%)	0.005**
Strabismus (%)	Orthophoria	40 (74.0%)	109 (86.5%)	0.047*	149 (82.8%)	179 (98.4%)	< 0.001*
	Esotropia	5 (9.3%)	11 (8.7%)		16 (8.9%)	1 (0.5%)	
	Exotropia	8 (14.8%)	6 (4.8%)		14 (7.8%)	2(1.1%)	
	Hypertropia	0 (0.0%)	0 (0.0%)		0 (0.0%)	0 (0.0%)	
	DVD	1 (1.9%)	0 (0.0%)		1 (0.5%)	0 (0.0%)	
	Total	14 (26%)	17 (13.5%)		31 (17.2%)	3 (1.6%)	
IOOA (%)	Yes	6 (11.1%)	10 (7.9%)	0.67**	16 (8.9%)	29 (15.9%)	0.27**
Pattern (%)	V	2 (3.7%)	5 (3.9%)	0.537*	7 (3.9%)	7 (3.8%)	0.84*
	A	0 (0%)	1 (0.8%)		1 (0.5%)	2 (1.1%)	
Ant. seg. anomaly	Corneal abnormality	0 (0.0%)	1 (0.8%)	0.507*	1 (0.5%)	0 (0.0%)	> 0.99*
	Cataract	0 (0.0%)	0 (0.0%)		0 (0.0%)	1 (0.5%)	
Post. seg. anomaly	Macula	4 (7.4%)	4 (3.2%)	0.35*	8 (4.4%)	4 (2.2%)	0.45*
	Periphery	2 (3.7%)	4 (3.2%)		6 (3.3%)	2 (1.1%)	

*BCVA* best corrected visual acuity, *LogMAR* logarithm minimum angle of resolution, *SE* spherical equivalent, *D* diopter, *DVD* dissociate vertical deviation, *IO* inferior oblique, *SO* superior oblique, *MR* medial rectus, *SR* superior rectus, *LR* lateral rectus, *SR* superior rectus, *IR* inferior rectus, *WTR* with- the- rule, *ATR* against- the- rule, standard deviation, *Ant* anterior, *Post* posterior, *seg* segment, *P* probability

^*^Based on Fisher Exact test

^**^Based on Chi-square

¥Based on independent T-test

Although refractive form of mirror image was observed in 5 (2.8%) of twins, there was not any strabismic form of mirror image in our study. (Right eye deviation in one child and left eye deviation in the other child.)

Table 3 shows the association of gestational age with other factors. Univariate analysis showed the significant correlation of low gestational age and female gender, low birth weight, neonatal jaundice, physical delay, seizure, amblyopia, and posterior ocular segment anomaly whereas only some of them showed significant correlation based on the multivariate analysis including female gender, low birth weight and seizure.

**Table 3:** Association of the gestational age and different factors based on both uni- and multivariate analyses

Gestational age	Univariate		Multivariate		
	OR	*P*-value	*P*-value	OR	95% CI for OR
**Sex**	0.166	< 0.001	< 0.001	0.364	0.224 to 0.592
Monozygotic	0.038	0.474	-	-	-
**Birth weight**	0.314	< 0.001	0.046	0.287	0.084 to 0.98
Neonatal jaundice	0.228	< 0.001	0.129	0.688	0.425 to 1.115
Physical delay	0.104	0.005	0.411	2.067	0.366 to 11.677
**Seizure**	0.192	< 0.001	< 0.001	0.016	0.002 to 0.143
Amblyopia	0.151	< 0.001	0.109	0.577	0.295 to 1.13
Deviation	0.031	0.413	-	-	-
Ant. seg. anomaly	0.051	0.175	-	-	-
Post. seg. anomaly	0.086	0.021	0.676	0.586	0.048 to 7.179

*Post* posterior, *Ant* anterior, *seg* segment, *OR* odds ratio, *CI* confidence interval, *P* probability

There were 86 twins and four triple (12 children) totally 184 cases in twins group, four of them (2.1%) were passed away due to severe physical abnormalities at birth.

## Discussion

The twins and non- twins comparison studies are the best way for determination of genetic factors of ocular disorders. Refractive mirror image is one of the strong genetic similarity of twins in addition to their identical phenotype and sex. These studies can also detect environmental factors more obviously.

In the present study, epidemiologic and ocular findings of twins were compared with non- twins who were age matched. There were 30% monozygotic and 70% dizygotic twins by considering their phenotype and sex similarities. Thirty- six (66.7%) of monozygotic twins and 72 (57.1%) of dizygotic twins were female. Female to male ratio was approximately 2 folds in monozygotic and 1.3 folds in dizygotic twins, whereas this ratio was near 1 (female, 46.2%) in non- twins children. Other reviews did not stress on gender of their study subjects.

Amblyopia was higher in twin, in comparison with non-twin children, it could be due to their more hyperopia, and higher percentage of amblyopia was also seen in monozygotic twins compared to dizygotic ones although there were no significant difference between their spherical equivalent.

Its reason may be related to more premature cases in twins, (less gestational age (*n* = 56) and low birth weight (*n* = 22)), and history of strabismus surgery (*n* = 28) as the results of the study by Bornstein et al. [15] that reported more retinopathy of prematurity, myopia and strabismus in prematurity in twin pregnancies.

Five (2.8%) twins showed refractive mirror image, of them 3 (60%) were monozygotic and 3 (60%) were amblyopic, but no one was anisometropic.

Monozygotic twin prevalence is 2/1000 of the general population and only 25% of monozygotic twins shows mirror image (5/100000 of the general population) [1, 3, 4]. Therefore, few case reports of mirror image were found in the literature which most of them had anisometropic refraction and were amblyopic [5] as ours. Some of other case reports presented strabismic mirror image by defining right esotropia in one child and left esotropia in the other child of the same twins [1, 6], but we did not see strabismic form of mirror image in our study. The reason could be due to different genetic factors in various societies and also due to our less sample size.

In our study, there were 2.36 folds more history of strabismus surgery of twins compared to non- twins with 4.5% attributed to being twins. In addition, higher percentage of strabismus was found in twins compared to non- twins especially in monozygotic individuals (*P* < 0.001).

In literature, parental smoking had significant association with strabismus as an environmental factor [7]. We did not have access to the data regarding the parental smoking of our study patients.

Although ocular anomaly, physical delay, seizure and macular hypoplasia were more in twins compared to non- twins, the difference was not statistically significant in our study.

Yu et al. [7] reported 38 out of 1000 birth anomaly on 20803 twins in California with more prevalence in monozygotic twins. Odds ratios were 5.91, 2.52 and 3.48 for clubfoot, strabismus and spina bifida, respectively.

Yu et al. concluded that birth abnormalities are multifactorial and both genetic and environmental factors could be effective together [7].

Thirty percent of our twins were phenotypically very similar, while phenotypic correlation were reported 68% and 40% among monozygotic and dizygotic twins in the study by Zhang et al [10], the reason could be related to the determination of the degree of appearance similarity which is a reliable matter. We did not study the similarity levels of central corneal thickness [11], optic nerve characteristics [8], based on the laboratory tests between twin and non- twins as Toh et al. [11] and He et al. [8] studies. The reason was the younger age of our cases for performing these tests in the most cases (central corneal thickness, optic nerve tomography), however, this could be considered as one of the limitations of our study. Additionally, determination of mono- and dizygotic twins only based on the phonotypic features without genetic testing might be considered as another limitation of this study.

Although being female, low birth weight, having neonatal jaundice, physical delay, amblyopia and seizure were found to have significant association with less gestational age in the univariate analysis, only being female, having low birth weight and seizure showed significant association with less gestational age in multivariate analysis.

## Conclusion

In conclusion, female sex, less gestational age, low birth weight, amblyopia, and strabismus were significantly higher in twins. Therefore, it is important to check their refractive error, amblyopia and strabismus to prevent their further complications.

## Data Availability

The data generated or analyzed during this study are not included in this article, but it would be available on request from the corresponding author.
